# Policy makers ignoring science and scientists ignoring policy: *the medical ethical challenges of heroin treatment*

**DOI:** 10.1186/1477-7517-3-16

**Published:** 2006-05-02

**Authors:** Dan Small, Ernest Drucker, Editorial for Harm Reduction Journal

**Affiliations:** 1 PHS Community Services Society, 20 West Hastings Street, Vancouver, BC, V6B 1G6, Canada; 2 Department of Anthropology and Sociology, University of British Columbia, Vancouver, BC, V6T 1Z1, Canada; 3 Montefiore Medical Center, Albert Einstein College of Medicine, Bronx NY 10467, USA

## Abstract

A decade of research in Switzerland, The Netherlands, Germany, and Spain now constitutes a massive body of work supporting the use of heroin treatment for the most difficult patients addicted to opiates. These trials concur on this method's safety and efficacy and are now serving as a prelude to the institution of heroin treatment in clinical practice throughout Europe.

While the different sampling and research protocols for heroin treatment in these studies were important to the academic claims about specific results and conclusions that could be drawn from each study, the overall outcomes were quite clear – and uniformly positive. They all find that the use of prescribed pharmaceutical heroin does exactly what it is intended to do: it reaches a treatment refractory group of addicts by engaging them in a positive healthcare relationship with a physician, it reduces their criminal activity, improves their health status, and increases their social tenure through more stable housing, employment, and contact with family.

The Canadian trial (NAOMI), now underway for over a year, but not yet completed, now faces a dilemma about what to do with its patients who have successfully completed 12 months of heroin and must be withdrawn from heroin and transferred to other treatments in accordance with the research protocol approved by Government of Canada, federal granting body and host institutions. The problem is that the principal criterion for acceptance to NAOMI was their history of repeated failure in these very same treatment programs to which they will now be referred.

The existence of the results from abroad (some of which were not yet available when NAOMI was designed and initiated) now raises a very important question for Canada: is it ethical to continue to prohibit the medical use of heroin treatment that has already been shown to be feasible and effective in numerous medical studies throughout the world? And while this is being worked out, is it acceptable to require patients who have been successfully treated with heroin in Canada, to be forced to move back to less effective treatments (treatments that failed to be efficacious in the past)?

This essay discusses this dilemma and places it in the broader context of ethics, science, and health policy. It makes the case for continuation of the current successful patients in heroin treatment and the institution of heroin treatment to all Canadian patients living with active addictions who qualify.

## 

"laws and institutions must go hand in hand with the progress of the human mind. As that becomes more developed, more enlightened, as new discoveries are made, new truths discovered and manners and opinions change, with the change of circumstances, institutions must advance also to keep pace with the times. We might as well require a man to wear still the coat which fitted him when a boy..."

Thomas Jefferson[1]

The Portland Hotel Society (*PHS*) is a non- profit social, health, and housing agency based in Vancouver's Downtown Eastside that has been active in providing services for people with active addictions for 15 years. The PHS operates a community based medical clinic treating this population with methadone maintenance, residential programs, and other approaches appropriate to people living with chronic heroin addiction. Despite the best intentions of these programs and the many fine clinicians practicing traditional addiction medicine, many in this population return again and again to heroin injecting – even in the face of the very real risks of AIDS, Hepatitis, overdose and the inevitable arrests and imprisonments associated with the illegal activities needed to get money for their habit. While many heroin users are eventually able to manage their addiction, some of the most troubled and persistent ones continue a downward slide and their continued use of heroin brings disastrous consequences. So obvious is this aspect of the heroin problem that many have said (including some police): "why not just give them the drug they seek – heroin?" Why not indeed?

On 29 January 2001, *PHS*, applied to the Federal Government of Canada for legal permission to prescribe heroin in Vancouver. Heroin prescription for the treatment of pain has been allowed in Canada since 1985 [2] and has been in the UK and many other countries for decades before that. Heroin maintenance of addicts has been used in clinical practice in the UK since 1926 [3]. Today, there are approximately 400 individuals receiving heroin in England as part of addiction treatment [4]. But Canadian federal regulators held that heroin treatment of addiction was a new use of the drug and that a Canadian research study would have to be done before any agreement to allow the use of heroin in Canada to treat heroin addiction. Assumedly this study was to be done for the purpose of assessing heroin's suitability for institution as a routine treatment in Canada, i.e. a first step towards making it routinely available for those heroin addicts who fit a profile indicating that it would be helpful for them in the treatment of their addiction.

Similar to the care of many cancer patients, the heroin treatment of opiate addiction isn't necessarily curative (i.e. having a goal of eventual abstinence from the drug); but is the best treatment option that the clinician can offer. In some of the saddest cases, it is essentially a palliative intervention aimed at reducing pain and suffering. But just as people living with cancer cannot simply enter the local pharmacy and purchase restricted drugs used in chemotherapy by medical oncologists in the treatment of cancer, so too, no one envisaged intractable heroin addicts going to the local pharmacy to purchase heroin. As patients in such a program, heroin addicts would need to be properly assessed by a qualified clinician and be able to receive the otherwise restricted treatment under vigilant medical supervision. And in order to properly supervise this form of treatment, Canadian practitioners would have to learn how to do it first hand. The start of this process has been the North American Opiate Medication Initiative (NAOMI).

The research team, led by principal investigator Dr. Martin Schechter, an epidemiologist and Director of the Centre for Health Evaluation and Outcomes Sciences (CHEOS), established Canada's leadership position in North America with respect to innovative addiction research. Structured from the outset as a rigorous randomized clinical trial (RCT) with very careful attention to research design and the selection of the study population and controls, the study was expensive by research standards, over CA $ 8,100,000 for 5 years, and one of the largest grants ever awarded by the Canadian Institute for Health Research (CIHR). However, viewed relative to the costs of illicit drug addiction to individuals, families and society (estimated at $48 per capita 1.4 billion Canadian dollars per year) NAOMI could prove to be a real bargain. [5]

The plan for the NAOMI was to recruit 157 experimental participants and an equal number of controls from three Canadian cities: Vancouver, Montreal and Toronto. Toronto later left the study and Vancouver and Montreal increased their study populations. As in other trials, the experimental group would receive pharmaceutical heroin (for 12 months) while the control group would receive methadone [6]. After nearly a decade of careful planning along with immense effort at the regulatory and funding level, the NAOMI began admission of its first patients in March 2005.

Importantly, even if the study showed positive outcomes for its participants no provision was made for continuing heroin for the study subjects after the 12-month heroin treatment phase of the experiment. This is not to say that the patients on heroin would be abandoned after one year. Under the study protocol, researchers planned to switch those subjects into methadone maintenance programs or other treatments of their choice. Recall that these same subjects were selected for NAOMI exactly because they had repeatedly failed at methadone treatment (or other treatment programs) in which they had not succeeded – including repeated attempts at abstinence in traditional treatment programs [6]. The ethical review boards at the three sponsoring institutions, the University of British Columbia, Toronto Centre for Addictions and Mental Health, and Universite de Montreal approved the study and its plans to transition the experimental group into the very same set of treatments conditions that had repeatedly been shown *not *to benefit them, regardless of the experimental results [6]. Thus while the NAOMI might help to establish a new standard of care and, hopefully, of *caring *for people living with active heroin addiction, the lack of an ethically sound pragmatic strategy for continuing heroin prescription if and when justified (based on all the available research evidence) was a worry from the outset [7].

The investigators for the NAOMI were trailblazers exploring new territory within the evolving framework of Canadian drug treatment policies based on harm reduction who were moving forward in the only way available within the regulatory framework. They could neither presume to know the results of their experiment before the science was completed, nor like previous studies in Switzerland and the Netherlands could they presume that the regulatory body would grant either an extension or expansion of the research exemption allowing heroin treatment for a time-limited period to a small group of addicted persons after the trial period. To insist that Canadian health authorities do so might have likely resulted in refusal of funding and the necessary authorization to complete the research.

Despite these complicated and potentially compromising circumstances, the researchers directing NAOMI did not blink as they undertook the political challenge to employ science to show Canadians a way out of the addiction wilderness–in the classic framework of rigorous clinical research. As public health and medical pioneers, they agreed to draw on their symbolic capital and accumulated prestige (see [8]) of their medical positions to discover the scientific answers that were required to build both the scientific and the cultural railway towards formal consideration of the institution of heroin treatment in Canada.

The NAOMI's formal task was to answer specific research questions about the feasibility, operational details and procedures, and a set of important clinical outcomes of heroin provision under medical auspices. But, from the outset, this research initiative was also implicitly and explicitly charged with the responsibility of helping to develop a medical solution to one of the most vexing problems facing Canada and many other nations, i.e. how to deal effectively with the persistence of illicit heroin injecting. Just as the problem was not a new one, there were also precedents to draw upon for its solution. Several other countries had gone down the same path of conducting careful experimental studies of heroin maintenance as a first step to instituting it as an important treatment option for the most refractory cases. What had they learned? How might Canada benefit from the results of these studies and experiences of these other societies as it strove to add another layer of competency to deal with its own heroin problems?

## NAOMI and her sisters

There have been several carefully controlled trials of heroin maintenance that came before the NAOMI including those performed in Switzerland (from 1995) the Netherlands (2000) and Germany (2002). These studies of heroin treatment varied with respect to the details of the research protocol and inclusion criteria for subjects (and accordingly each measures somewhat different outcomes in different ways). But each of the European trials was a test of the same basic proposition – could heroin be used in medical practice to engage a target group previously not reached or failed in a clinical relationship? And could it reduce biological, social and personal risks of injecting illegal drugs while reducing or eliminating crime by necessity and survival sex trade work for active heroin users?

There are benefits to studying the use of a drug in different settings and the NAOMI was not an exact re-run of previous trials. The NAOMI's particular clinical criteria and protocol were adjusted for the Vancouver and Montreal population: the subjects were of course all chronic heroin users – and over 40% with HIV. They were all individuals who had failed in many other attempts to bring their dangerous and costly addiction under some control. The study focused on this local sample as representatives of the hardest cases of a much larger population (between 60,000 and 90,000 in Canada according to CIHR with some estimates as high as 125,000) desperately in need of new and more effective models of care[6,9].

The intravenous drug using population in Vancouver includes both those addicted to heroin as well as those addicted to cocaine and other stimulants. For instance, close to 5000 individuals registered to utilize Vancouver's supervised injection facility (SIF). Of the injections taking place at the SIF, approximately 46 % are heroin, 37 % cocaine [10]. Those opposed to harm minimization public health approaches, sometimes make the argument that Vancouver's addicts are substantively different in terms of the substances they inject in their bodies, and the impenetrability of their addiction, and that this somehow justifies a more guarded approach to innovations in addiction medicine. Addiction is by its very nature challenging, in all jurisdictions, and Canada does not have the market cornered on the hardest to treat of those living with addictions. There are opiate, stimulant and polydrug injectors in many countries including the Netherlands [11], Switzerland [12], Australia [13], Spain [14], the United Kingdom [15], as well as Germany, Italy and Ireland [16].

Further, in addition to the European trials, there was nearly a century of clinical practice and experience in the UK where physicians prescribed heroin for both pain management and addiction in routine practice. This work in the UK was not initially set up as a study, but was a repository of decades of practical clinical experience with heroin treatment for the UK's general medical practitioners, who, despite some restrictions always had the legal right to exercise their clinical judgment with respect to prescription of heroin. Heroin prescription occurs in a wide variety of clinical settings in the UK (in several areas of England as well as Scotland and Wales) there have also been four previous studies of heroin prescription – in the 1970's, 1980's, 1990's, and 2001 [17] – and another is underway now. This fundamental clinical work in the UK shows that it is feasible to maintain some addicts on medically prescribed heroin, even for decades, and still sets important precedents for understanding the regulation of heroin treatment in specialty addiction medicine practice. But what did the rigorous research from other countries show that could guide Canadian research and policy decisions?

### Switzerland

The Swiss were the first to show these effects through a careful evaluation of prescribed heroin for over 1,000 of the countries most refractory, long-term heroin addicts – targeting the most difficult of individuals who have had long-term difficulties with substance misuse and repeated failures with traditional abstinence based approaches to treatment. The Swiss studies showed unequivocally that prescribing heroin produces substantial declines both in illicit drug use and in criminal activity for this most problematic group. In addition, they provided clear evidence of improved social reintegration, i.e. better housing, more gainful employment, fewer drug associates and more contact with previously estranged families and friends. Here are some of details:

• *Fitness for work improved considerably: permanent employment more than doubled (from 14 to 32%), unemployment fell by more than half (44 to 20%)*

• *The patients' housing situation rapidly improved and stabilized (there was in particular no homelessness)*

• *There was no fatal overdose due to prescribed substances*

• *No notable disturbances in local neighborhoods*

• *Significant economic benefit in terms of savings per patient-day (relating to savings in criminal investigations and prison days, followed by improvements in the state of health of the participants)*

• *There was a marked decrease in shoplifting (35% to 16.1%), breaking and entering (6.9 % to 0.0%), drug dealing and handling stolen goods (13.1% to 3.9%), sale of hashish (26.3% to 12.5%), sale of hard drugs (46.9% to 8.2%) based on interviews at time of admission and after 12 months of treatment*

• *Overall, offences dropped by 68%: Notably, this drop is not limited to short periods of time. The data from the Swiss study shows that this drop remained stable after 24 months of treatment. According to the Central Criminal Register, the number of convictions dropped by 80% *[18]

The Swiss approach to heroin treatment has been criticized for being a program initiative rather than a randomized trial. But the RCT is NOT the only means to determine efficacy of new treatment strategies–indeed in public health programs it is rarely even an option. The successful introduction of methadone treatment occurred without one, likewise the widespread use of penicillin following World War II.

The Swiss health authorities desired to move ahead quickly with a heroin project that was as much demonstration and proof of principal as it was research *per se*. But many of methodological concerns associated with their approach were addressed (in advance) by the Swiss investigators, who went to great lengths to be conservative in their methods and cautious in the conclusions they drew from their results [19]. All the limitations of the study design were well recognized and repeatedly addressed by the meticulous Swiss investigators and in no way diminished the significance of their landmark study that revealed that alternatives to methadone maintenance can attract and retain addicts for whom methadone has proved unsatisfactory [20,21]. The Swiss study was a response to an epidemic – its heroin injectors had the highest rate of HIV infection in Europe. Similarly, if a bird flu pandemic were to occur, governments would not utilize a RCT approach to test available public health intervention strategies. The Swiss trial highlighted the positive effects supporting a need to change policy and practice – that's why they undertook it in the first place – and the fact that its outcomes led directly to policy changes instituting heroin treatment in Switzerland, was the whole point of the enterprise, not a felicitous fringe benefit.

The fundamental premise of all narcotic maintenance is that, for many patients addicted to opiates, the use of any one of a number of substitutes produces health and psychosocial results far superior to illicit street use or drug free treatment. Drug abstinence was never the goal for the Swiss patients, their doctors, or the Swiss Federal Office of Public Health. The original intent of the study was to improve retention of the patients in care, show improvements in the clinical course of their addiction (i.e. reduced illicit drug use and high risk injecting), and specific gains in the social outcomes associated with a diminution in the use of illegal drugs.

The outcomes from this study that account for its public acceptance in a national referendum and the Swiss Federal Health Department's decision to extend and expand the program by over 50%, and for a half dozen other nations to express interest in replicating the Swiss work. The objective of reducing AIDS risk among the Swiss population was paramount to thinking about the use of research such as this in the context of responsible leadership in public health policy and is highly pertinent to the situation in Canada.

Furthermore, re-enforcing the success of this strategic use of research to inform both Swiss policy and larger concepts of the scope of professional practice in addiction medicine, even the preliminary Swiss results that were decisive in persuading Dutch public health officials to initiate their own randomized study of heroin prescription in Amsterdam and Rotterdam (for 750 participants), and formed the basis of support for conducting similar programs as randomized controlled trials in Germany and in Canada.

### The Netherlands

The Dutch Heroin Maintenance Study was considered the most rigorous study to date (it *was *an RCT) and its results are fully available today. [22] Again these studies showed that it was feasible to conduct a program that made heroin medically available (for a 12 month trial period) to a group of hard core addicts with multiple prior failures in treatment. It also produced very positive results:

*Adherence was excellent with 12 month outcome data available for 94% of the randomized participants. With intention to treat analysis, 12 month treatment with heroin plus methadone was significantly more effective than treatment with methadone alone in the trial of inhalable heroin (response rate 49.7% v 26.9%; difference 22.8%, 95% confidence interval 11.0% to 34.6%) and in the trial of injectable heroin (55.5% v 31.2%; difference 24.3%, 9.6% to 39.0%)." *[22]

But when the Dutch trial was ended and patients had to stop the prescribed heroin and switch to methadone, many reverted to heroin use and there were serious adverse consequences for the addicts:

*Discontinuation of the co-prescribed heroin resulted in a rapid deterioration in 82% (94/115) of those who responded to the co-prescribed heroin. The incidence of serious adverse events was similar across treatment conditions. Conclusions were that the supervised co-prescription of heroin is feasible, more effective, and probably as safe as methadone alone in reducing the many physical, mental, and social problems of treatment resistant heroin addicts." *[22]

We will return to a discussion (in a later section of this paper) of this crucial problem in all heroin research – i.e. how to terminate the study when it is successful.

### Germany

In Germany, approximately 35,000 to 40,000 heroin addicts are in methadone treatment and about 35,000 are not in any treatment [23]. The heroin study was a multi-site trial in the cities of Bonn, Karsruhe, Koln, Hannover, Frankfurt, Munchen and Hamburg. It is, like NAOMI, a randomized control study that focuses on two target groups: those heroin addicts presently enrolled in methadone maintenance that have not made significant gains in treatment and those that are not yet in medical care but require it due to their health status or life circumstance.

The main purpose of the study was to compare injectable heroin to oral methadone with respect to treatment outcomes in health, level of illicit drug use, crime, treatment retention, disengagement from drug community, employment, social connection, housing situation and ability to reach a difficult target group [24]. The total number of participants in the study was 1,120 with 560 in the methadone group and 560 in the untreated group. The first participant was enrolled in March 2002 in a randomized control trial to examine clinical efficacy of heroin in a structured treatment setting as well as its impact on crime, healthcare, cognitive-motor and neuropsychological functioning [25]. A cost benefit analysis and evaluation of the effects of two psychosocial interventions (motivational interviewing and addiction counseling) was also a part of the trial. The pharmacological component of the study was designed for 24 months with the entire study taking 36 months.

The results were "unequivocally positive" was the conclusion reached by Federal authorities and those of all seven participating cities where illegal drug use was reduced, health status improved, and there was better social integration and less criminality [26]. In light of these results, the continuation of heroin treatment (as clinical practice in Frankfurt) was approved by a large majority of the Frankfurt City Council ("Stadtparlament"). The approval of heroin as prescribed medication for all of Germany is expected by June 2006. Based on the favorable results, prescribable medication for the treatment of addiction, heroin treatment for addiction as part of standard medical practice has been recommended by the drug police coordinator for Germany, Caspers-Merk [27].

### Spain

With approximately 150,000 people living with serious and persistent heroin addiction, three autonomous regions of Spain (Andalusia, Catalunya and Basque County) took the lead in convincing the Federal government to provide the legal framework to allow clinical trials of heroin prescription in 2001 [28,29]. From the inception of the research, the trial's researchers publicly disputed the Federal government's criteria that the patients would have heroin withheld at the end of the trial. Instead, the health authorities from the autonomous regions and the principal investigators took the firm position that it was medically unethical and inhumane to cut patients off clinically prescribed heroin once the research protocol was completed [30,31].

While the Basques have not yet been successful in obtaining final approval for their proposed research trial from the federal government of Spain, both Catalunya and Andulusia have completed trials examining the administration of heroin for addiction [28]. The Catalonian research compared oral methadone therapy to oral heroin therapy while the Audalusia randomized trial compared intravenous heroin to oral methadone as medical treatments for marginalized people addicted to heroin that had not been successful in other treatments [32,33]. A peer-to-peer recruitment strategy was employed as a strategy for enhancing the researchers ability to reach the target group.

Those prescribed heroin in the experimental group made more gains than the control group receiving methadone with respect to health and also demonstrated a corresponding decrease in criminal activity, illicit heroin and cocaine use, drug related problems and risk behaviors that might lead to HIV infection [28]. The Andalusia research took place between February 2003 and November 2004 and the results are presently being evaluated by the Spanish Drug Agency. The full results of the trial are due to be published shortly [34].

## Lessons learned

While these sampling and research protocol differences were important to the academic claims about specific results and the general conclusions that could be drawn from each study, the overall outcomes were quite clear – and uniformly positive. The cumulative international research and the massive body of work to date all point to the finding that the use of prescribed pharmaceutical heroin does exactly what it is intended to do–to reach a nearly unreachable group of people by engaging them in a healthcare relationship with a physician; reduce their criminal activity; improve their health markedly; and increases their social tenure in terms of homelessness and employment.

While there are some differences, all of these studies and their objectives, especially Germany's heroin project, are very similar to NAOMI in several important ways – the treatment population, sampling criteria, and research designs that compare heroin to other treatment options. The study objectives of these trials all closely parallel those of the Canadian trial – to assess the suitability of prescribed heroin for addiction treatment. And all the heroin maintenance trials target difficult to treat, socially marginalized and unwell individuals living with serious and persistent addictions. The unique characteristics of each locality is not a justification for repeating randomized control trials perpetually when the clear and obvious beneficence of a medical treatment has been shown across scores of settings. This would not be so easily justifiable in other medical trials such as in the instance of cancer.

The existence of their results now raises a very important question for Canada. Is it ethical to continue to prohibit the medical use of a treatment that has already been shown to be feasible and effective in numerous well controlled scientific studies with similar populations of heroin users seen in a wide range of clinical settings similar to what is available in Canada?

## Canada at the cross roads: evidence and ethics

A decision point is about to be reached for the use of heroin treatment in Canada. This is precipitated by the looming expiration of the 12-month trial period built into NAOMI, where a few dozen individuals are already receiving heroin treatment. According to the research protocol, those patients who have been maintained under the experimental regime must leave it after 12 months. A growing number are scheduled to have heroin treatment withdrawn within the next few months. This is not an academic issue. In light of the evidence of feasibility of operating the program safely already available from NAOMI, and all the other trials, is it ethical to continue on the present course? Do we really want to say that in Canada patients who are doing well in a medical study can only continue to have access to life saving treatment (which has been shown repeatedly to be efficacious elsewhere) through their continued participation in time constrained research trial?

We know that some patients in heroin treatment use it as a pathway to reducing or stopping their heroin use altogether. In several of the European studies, some individuals chose to switch to methadone (usually in combination with program heroin) while still on the trial. Using sufficient doses, they generally do well (at least in terms of staying in treatment and not relapsing to heroin). Some even went into abstinence treatment in Switzerland. But in the Netherlands, most did not want methadone and it is likely that will hold true in NAOMI as well. It is noteworthy that in the Swiss trials and often in UK practice, once individuals in heroin treatment stabilize their lives, they often prefer the less onerous program of methadone with weekly or even monthly visits to clinic, rather than thrice daily for heroin. This demanding regimen offers little chance for a normal life, but is still better than having to engage in risky activities such as crime or sex trade to gain enough income for daily dosages of illegal heroin.

The Dutch have recently presented more detailed data on what happens to people ejected from heroin treatment because of the 12-month time restriction that was built into their trial (as in NAOMI). In the Dutch heroin trial, participants were considered to be "treatment responsive" if they showed a 40% improvement in at least one domain (e.g. drug use, arrests, health and mental status) where they performed poorly at the beginning of the study. Most did well. Of the group of 55 participants that completed treatment during the first year of the experimental period, 32 (58.2 %) were considered to be treatment responsive. And, as is proposed for NAOMI, even patients doing well were made to stop heroin treatment and either go onto methadone, drug free programs, or leave treatment altogether.

However, in the Dutch study a compassionate care provision was built in for safety – allowing the investigators to re-admit individuals to heroin treatment if they reverted to heroin use after the study. And most did, rather quickly. Of the "successes", 84% deteriorated within two months after heroin treatment was discontinued [35]. But most of these were re-admitted to the program, and the Dutch are now planning to institute the program as part of the available repertoire of treatment options.

The dilemma is clear – for many of these subjects (also still *patients*) who have responded well to NAOMI, stopping the provision of heroin will throw them back into a world of pain and high risk as they, predictably, strive to self-medicate with impure and unregulated illegal versions of heroin they can no longer receive safely under the watchful eye of a NAOMI physician. This challenge continues as a growing crisis for NAOMI and its subjects as more patients near the 12-month point.

## Research ethics and public health

The most widely accepted document outlining ethical standards for research at the international level is the Declaration of Helsinki [36]. There is a crucial section, paragraph 30, of the document that is pertinent to research on heroin treatment for addiction. It reads:

"At the conclusion of the study, every patient entered into the study should be assured of access to the best proven prophylactic, diagnostic and therapeutic methods identified by the study"[37]

The main motive for this portion of the international research guidelines is to prevent the sponsors of research trials (government, university, hospital or private) and physician collaborators from initiating research on subjects who would otherwise be unable to access the treatment offered in the research and then taking away the treatment when the research schedule is complete [36]. Similarly, the International Code of Medical Ethics of the World Medical Association (WMA) has recognized since 1949 that: "a doctor owes his patient complete loyalty and all the resources of his science"[38].

The NAOMI has strictly fulfilled the ethical requirement of the Declaration of Helsinki, particularly with regard to the clarifying footnote added to paragraph 30 by the World Medical Association in 2004:

"The WMA hereby reaffirms its position that it is necessary during the study planning process to identify post-trial access by study participants to prophylactic, diagnostic and therapeutic procedures identified as beneficial in the study or access to other appropriate care. Post-trial access arrangements **or other care **must be described in the study protocol so the ethical review committee may consider such arrangements during its review [emphasis added]."[37]

The NAOMI definitely identified post-trial arrangements and the ethical review boards of three institutions definitely considered these arrangements in their review. However, the regulatory framework with respect to heroin treatment for addiction left the research designers and ethics reviewers in a difficult position of only being able to offer, legally, already available traditional treatments that had already repeatedly failed the patients in question. In a difficult ethical position from the beginning, they daringly chose to carry onward because there was simply no other option available to them if they were to stand any chance to lay the groundwork for heroin treatment in Canada. Without the clarifying footnote added to paragraph 30 of the Declaration of Helsinki by the WMA in 2004, the NAOMI would not have been able to move forward ethically.

Although this is commonly disregarded in the scrum of international drug licensing differences and regulatory variations – i.e. many well respected medications in long term use in Europe are unavailable in the US, or items available over the counter in one country, are available only with prescriptions in another – in this case it is not profits but human lives that are at issue. The scientific facts about many key addiction initiatives are not really in question. What is in dispute is how governments, in this case the Government of Canada and its regulatory bodies, should respond to the evidence base that science has by now produced in some abundance, and the implications of that evidence for practice in Canada.

Addiction treatment policies, like any medical or public health practice, should be evidence-based. If not driven by evidence they always have the potential to lose their moral compass, especially if effective treatment that is available is withheld. The infamous US Tuskegee Syphilis Study provides a sad historical case of such treatment being withheld, in this case penicillin, that was developed after the trial began, but not made available to participants for the sake of completing the scientific study. While it may make some uncomfortable to relate this historical case to the present situation, we present Tuskegee to make the point that well-intentioned people who believe their actions to be in the best interests of people and society sometimes make unethical decisions that lead to social and medical disasters.

But closer to home in Canada, we need only look back to the quarantined lepers of Canada that were relegated to isolated islands, D'Arcy Island and Bentinck Island, off the coast of Vancouver Island to die without medical or social support when their disease was amenable to medical care. The lepers were given only rations and coffins and the Government of British Columbia rebuffed assistance from missionaries [39]. The medical establishment was aware that leprosy was not acutely contagious but in spite of this knowledge the panicked government officials exiled the afflicted and left them to die without care. The ailing lepers were expected to bury the dead until the banishment stopped in 1957 when the last person with leprosy died. Only lepers of Chinese ancestry were reduced in importance to receive this fate; the Euro-Canadian lepers from all across Canada were sent to receive the kind help and healthcare of the nuns of the Hospitallers of Saint Joseph in New Brunswick [40].

A further example is seen in the establishment of reserves or reservations for people of aboriginal ancestry, complete with "Indian agents", that historically dictated when cattle could be slaughtered, what crops were planted, where children were schooled and in what language. In Canada, it was not until 1960 that all people of aboriginal ancestry were allowed to vote in federal elections and they were not given this manifestation of personhood in Alberta provincial elections until 1965. Individuals that believed that they were helping aboriginal peoples in a relationship of tutelage designed these social initiatives [41].

The Tuskegee Study, commenced in 1932, was originally intended to take approximately one year, but continued uninterrupted for forty years. This included a period of some twenty-seven years after penicillin became the accepted and widely obtainable cure for syphilis. Even then, the discontinuation of the study was precipitated not by medicine or public heath officials but by an article by journalist Jean Heller published on the front page of the Washington Evening Star (25 July 1972). Twenty-five years later, on 16 May 1997, after hundreds of preventable deaths in the original study cohort and allowing the participants to unknowingly transmit the disease to their partners and children, President Bill Clinton formally apologized on behalf of all of America for the Tuskegee study and the role that the US government had played [42].

We do not mean to demonize research, policy makers or researchers. It is unlikely that either the researchers or policy makers harbour any ill will towards those living with active addictions living in the shadows of society. Similarly, those who initiated the Tuskegee study did so as part of a genuine dedication to improving quality of life for African American people [43]. Nor was the study intended to be ethnocentric. It was completed in partnership with African American physicians, researchers and administrators. The Tuskegee study of the effects of untreated syphilis is different in one fundamental way from heroin treatment for addiction element. At the time that Tuskegee study was initiated, there were not efficacious treatments for syphilis[44]. In contrast, in the case of heroin treatment, it is a clinical intervention that has an established scientific evidence base demonstrating its efficacy. The continued government regulatory handling of a clinical intervention, in this case heroin treatment, as a purely scientific question thereby limiting access by patients who would clearly benefit from it is, in our view, unethical and increasingly politically and medically untenable.

One of the challenges to the development of best practices in healthcare in Canada and elsewhere is the fact that controversial innovations, such as genetic testing or addiction treatment, are required to continue as research initiatives for scientifically unwarranted periods of time. These lengthened periods of time would not be acceptable in other clinical trials. In addition to the genuine uncertainties and questions that need to be answered before we can proceed with something new and potentially hazardous in medicine or public health, research is sometimes used as a way to buy time. But while it is a way to buy time during the period when the balance of public opinion about addiction treatment evolves, it is ethically untenable, in our view, to continue any treatment research (heroin included) when the research questions have been substantially addressed and answered in other jurisdictions.

## Consent revisited

As the NAOMI reaches the end of its first year, the first participants are due for discharge from the trial – that is what they agreed to when they entered the study in the first place. But that consent may need to be reexamined in the light of subsequent evidence. And the fact that heroin addicts can only receive the treatment they desire for a time-limited period that is dictated by research protocol, without recourse to (for them) an effective treatment that has proven itself in other valid studies, is (at best) ethically awkward with respect to informed consent. Modern standards of informed consent have been developed most substantially since the establishment of the Nuremberg Code for experiments involving humans established in 1946 as a result of the legal prosecution of Nazi physicians [45]. The expectation of informed consent for human subjects is a complex issue when it comes to marginalized individuals with active addictions whose access to the analgesics to which they are addicted is only available through research? It is, for example, disputable as to whether Canadian citizens addicted to heroin that can only receive medical grade heroin in Canada by participating in a rigorous scientific study are actually providing informed consent without duress.

## Harm reduction: evidence vs. politics

Consider the alternative to an evidence based medical and public health response to the problem of refractory heroin addiction. In the United States, incarceration is the most widely available "treatment" response, universally "available" to drug users. While jail is widely touted as a step to coming to terms with ones demons, all too often criminalization converts a treatable drug problem into a social and personal nightmare – a disastrous beginning point for clinical intervention. While treatment compliance for other chronic diseases varies, no one suggests that refractory diabetics or cigarette smokers or alcoholics should be incarcerated "for their own good". Yet those living with addictions often end up in the unwelcoming arms of the criminal justice system.

Despite the mass of decisive evidence on the efficacy of methadone in treating opiate addiction, methadone is still treated as a pariah drug in many parts of the world. Entire nations (e.g. Russia and India) today forbid its use in addiction treatment and, in the US (where methadone was first employed for addiction treatment) eight states still outlaw its use. Even in the United States, though medically approved, the clinical application of methadone is so irrational (e.g. justifying systematic use of sub-clinical doses) or downright mean-spirited and punitive, that, despite its proven efficacy, many have grown to hate it. Indeed much of the allure of the newly approved use of buprenorphine is that it is "NOT methadone".

So important is the predominant demand reduction approach in the United States that the Director of the Office of National Drug Control Policy, Mr. John Walters, has budget certification authority so that he can decertify the budget of any federal department if it does not meet drug control policy objectives. The Director is a cabinet position that is appointed directly by the President. Under this department, as part of the Executive Office of the President, there are 4 additional Senate confirmed positions that all report to Mr. Walters. The Director reports directly to the President of the United States and reviews all budgets before they are finalized [29]. Accordingly, needle exchange and distribution is widely accepted within the US as scientifically irrefutable, yet almost universally disregarded and denied funding by federal and many state and municipal authorities.

To learn more about this first hand, one of us (DS) was part of a group of international experts and practitioners in substance abuse health programs who visited the US in November of 2005, under the United States International Visitors Program of the US Dept of State. On one leg of that trip the group visited the Bloomberg School of Public Health at John Hopkins University in Baltimore, where several lecturers provided an overview of their research. They emphasized the importance of science and, in particular, epidemiology in understanding addiction. When asked about the science on needle exchange which US Federal policies does not allow or support one of the lecturers resolutely stated to the group that they [he and his fellow faculty present in the room] "know that needle exchange is efficacious in saving lives but that it will never receive public funding for political reasons".

Later, the group visited the National Institute on Drug Abuse (NIDA), the research-funding arm of the Government of the United States (whose billion-dollar budget funds more than 85% of all the addiction research in the world). When asked about needle distribution, the epidemiologist and ethicist for NIDA promptly asserts that they (presumably the senior scientists of NIDA) agree that needle exchange is efficacious in preventing HIV and HCV infections "but we are not policy makers". Another senior administrator and research psychologist for NIDA added to the discussion by making the point that " drug users do not have an organization or lobby so they have no impact on policy". The approach of the US federal government to needle distribution provides a current example of policy makers ignoring scientific evidence for efficacious population health intervention: with disastrous results.

## Beyond randomized control trials

In light of the now massive body of scientific evidence supporting the efficacy of heroin treatment with thousands of patient years of experience in 5 national research trials with the Swiss having prescribed heroin to several thousand since the mid 1990's, a few hundred in the Netherlands and Germany and over 75 years of UK general medical practice–the PHS followed up its original request to Health Canada with an appeal on 2 January 2006 asking once again that community physicians in Vancouver's Downtown Eastside be permitted to prescribe heroin through a clinic for people living with active addicted to heroin but who have been unsuccessful with other clinical interventions. This appeal was made to Canadian health authorities on medical ethical grounds, asking for an exemption under Section 56 of the *Controlled Drug and Substances Act *(CDSA).

In a response written 14 February 2006, the Department of Drug Strategy and Controlled Substances for Canada stated that:

"While heroin prescription has shown some promising results in Europe, sound evidence is needed to demonstrate its effectiveness in the Canadian context before Health Canada can be in a position to exempt heroin prescription for medical purposes..."

The letter points to the NAOMI study goal to determine whether heroin treatment:

"...Will improve the health and quality of life of injection drug users, reduce homelessness and decrease their interactions with the criminal justice system."

Just because the patients are addicts and the treatment is heroin, this issue for NAOMI does not exist in an ethical vacuum. We believe there is a medical ethics legal argument to be made regarding standards of care and access to effective health services for people with active addictions.

If the research in another sector were as clear, this treatment protocol would by now be available e.g. if a new drug for breast cancer or colon cancer were shown to be as efficacious and effective as heroin has been shown to be, then the clinical trial would generally be stopped and the medical program, with ongoing scientific evaluation, would commence immediately. If this were another drug trial, say for treating hypertension, would there even have been a statutory requirement for a study to be repeated in Canada that had already established efficacy with thousands of patients, and whose results had been published in peer reviewed scientific literature elsewhere? While replication is an important part of the scientific method, does a scientific fact have to be repeated in every single jurisdiction? Would the prescription of a drug shown to be effective in treating breast cancer in Switzerland the UK and The Netherlands have to be replicated in Canada before it would be made available for Canadian patients? Extending this logic further, would the very same study also have to be replicated in each of the individual provinces within Canada to verify that the findings were applicable equally in differing contexts of Newfoundland and Alberta?

We cannot choose to use evidence only when it suits policy objectives and ignore it when it contradicts them. Nor can we discourage the collection of evidence base, using scientific methods, for initiatives that are politically unpopular such as supervised inhalation rooms. Once science has demonstrated the evidence-based outcomes for an efficacious medical treatment, then governments have a medical legal obligation to citizens and prospective patients to grant the legal authority to practitioners to provide them as part of the continuum of available treatments. The Government of Canada through the Department of Drug Strategy and Controlled Substances at Health Canada needs to grant a medical exemption for heroin treatment immediately so that patients who would benefit from this medical treatment have access to it in order to better live their lives.

The dilemma facing the NAOMI with respect to its subjects at the end of the first phase of the trial has shown us something very important: harm reduction is itself at a crossroads. The potential for the medical prescription of heroin is not alone at this busy, and dangerous, intersection of science, politics, ethics and morality as applied to drug use and harm reduction. In a further letter to Health Canada, the PHS requested permission to go beyond the Supervised Injection Facility and operate a supervised inhalation room – for people addicted to crack cocaine and crystal methamphetamine in Vancouver's Downtown Eastside. The agency already operates the Supervised Injection Facility and perceived an Inhalation Room as a natural extension of this harm reduction initiative. The request was denied, stating that evidence was required to prove that an inhalation facility could meet health needs. The science, according to Health Canada, did not exist at present and furthermore, an argument would have to be made that such a facility would benefit from scientific examination. So, then, the Director General of the Federal Department of Drug Strategy and Controlled Substances essentially asserts that science doesn't exist and such a research question might not be worthy of scientific study anyway. While relying on science to deny the request, the Director makes a final fascinating assertion that an exemption for a scientific study of a supervised inhalation room would need one final requirement:

"The demonstrated support of key stakeholders and partners, such as municipal and provincial health authorities and law enforcement agencies."

(Correspondence from Health Canada dated 1 February 2006)

Here, the Health Canada representative makes it a condition that to obtain permission to initiate a scientific research study one would require support from municipal and state politicians along with that of state and federal police. It is difficult to imagine another parallel where a scientific research study, such as a clinical trial for a treatment for colorectal cancer, would require the study's authors or principal investigator to obtain a written endorsement from the Chief of Police, Mayor and City Council and Provincial Health Minister to proceed. It appears that the scientific method is being used as part of a government risk management strategy [46]. In the case of institutional risk management, the perceived risk is to the institution–a political risk–and the focus is on protecting the government and not the patient. Indeed it could be argued that the actual risk management issue for the Government of Canada in the case of heroin treatment is in failing to act when the scientific evidence demonstrating an efficacious treatment already exists.

## Conclusion: a time for action

Our goal has been to make the case that when it comes to addiction treatment, policy makers sometimes ignore science and scientists sometimes ignore policy. This mutual failure can be very costly. Science is but one arrow in the quiver of policy change, but it is the responsibility of a human being, acting ethically, to choose the arrow, and to decide whether and when to release it from the bow and in what direction to aim it. The NAOMI arrow is in the air, but where will it land?

While medical research is certainly important for examining the efficacy and effectiveness of new treatments there is more at stake in Canada's NAOMI than simply scientific goals. Scientific results alone may not be sufficient to affect practical policies, especially in the case of providing a drug as demonized as heroin to addicted patient's, and calling it treatment.

The Canadian heroin trial, like those that have taken place in Switzerland, the Netherlands and Germany, addresses a perplexing, expensive, and epidemiologically dangerous dilemma: either severe addiction, intractable and destructive, is an illness, or, it is not. There are those who see great threat in all drug maintenance approaches, believing that some moral ideology they hold, rather than clinical outcomes, should determine medical practice. This is a dangerous idea and must be confronted head on. So the conflict over heroin maintenance is also a great opportunity, a chance to take an important step forward in the medical responsibility and competency to engage the world's growing problem with heroin injection, now driving the AIDS epidemics of dozens of countries and millions in population.

For much of the world, drug use and addiction are still shrouded in a medieval cloak of moral disapproval. And many nations' policies are still punitive and unforgiving of professionals who are too accepting of drug use – witness the sharp attacks on Harm Reduction in the US and in UN drug policy bodies [47]. With the pervasive and easy access to potent drugs of all sorts growing daily (especially for the worlds poor and most disenfranchised populations) and the paucity of access to effective treatment, we have so far managed to turn addiction from a treatable condition into the public health nightmare that has relegated millions of people living with serious addictions to poverty, prison and early death from preventable infections.

Basic non-judgmental harm reduction ideas, almost universally seen as supportive by drug users, their families, and human rights advocates, are rejected by many authorities as "sending the wrong message" (vs. zero tolerance), as "enabling" by hardliners. In addition, the notion of "drug legalization", removing the drug issue from the realm of criminal law altogether, is used as an accusation akin to treason. And sadly, this is also true of addiction treatment in much of medical practice. Various forms of opiate maintenance treatment for serious and persistent heroin addiction already have a better prognosis than many other chronic medical or psychiatric conditions. Yet clinical and scientific ignorance, therapeutic nihilism, and medical neglect are still the norm in most of the world.

While genuflecting to the need for evidence base is part of the new high mass for policy makers and addiction treatment providers, in the case of addiction, the larger evidence base of population data is often ignored. Across the globe, most addiction treatment policy and practice have only the most tenuous relation to scientific evidence – witness the persistence of moralistic approaches based on self abnegation, religious conversion, or tough love with hardly any evidence to support them.

Addiction is a complex social issue that demands complex social solutions. The modernistic employment of science by the Canadian government to unearth incontrovertible answers to addiction is misguided. Research, in contrast to science, begins with recognition of the messiness of the constellation of issues surrounding social issues like addiction. Scientists, politicians and activists should not "expect science to decrease the complex web of their lives" [48].

## An exit strategy

The focus for the transition plan regarding heroin prescription under NAOMI should now shift from the patients to the trial itself. The NAOMI researchers have served Canada well and boldly carried their trial as far as they can in difficult legal and regulatory circumstances over which they have no control. The researchers have shown strong leadership; and now it is time for policy makers to meet them halfway and liberate them from the dilemma of a study protocol that will force them to withhold treatment that they now know is feasible, well accepted, safe, and effective for their patients.

The original grant dollars provided for the research should be supplemented by the provincial and federal governments of Canada in order to expand the scope of the NAOMI into a full scale addiction treatment program with the present researchers overseeing ongoing evaluation. The implementation of a full-scale heroin treatment program could result in millions of dollars in cost savings in public funds in Vancouver [49] and billions [50] in Canada due to a reduction in hospital days, visits to emergency departments and involvement in the criminal justice system, in addition to an increase in employability.

The trademark of high science, the randomized control trial, will not unshackle policy makers and politicians from the responsibility of making the difficult political decisions that are steadfastly rooted in the social world. Today, if ever, science itself does not meet its own "high" standards for disconnection from society, and even virtuous science is not really chaste – a pure science unfettered by the vagaries of society. Witness the contemporary scandals in pharmaceutical and stem cell research.

We know that the realms of science and the social are not really separate spheres:

"The adjective 'social' has been used to weaken science's claim to truth and certainty. And if you say that science is socially constructed, that is considered wrong by scientists. This tug-of-war between science and society, where one gains what the other loses, is not longer the only game in town. There is now an alternative. To the old slogan of science–the more disconnected a discipline from society, the better–now resonates a more realistic call for action: The more connected a scientific discipline, the better."[48]

All illness has a social component. The social dimension is crucial for effective medical treatment and is clearly visible, if one stops to look, in the etiology of disease as well as the quality and significance of its lived experience for the patient, their family and the community. This is true whether it is cancer, diabetes, schizophrenia, AIDS or addiction.

In the case of heroin prescription, the significance of the social context does not negate the unique place of the physician, who alone has the societal authority and responsibility to prescribe this highly restricted drug to treat disease and alleviate otherwise insurmountable pain. If medical practitioners do not shoulder their responsibility in this matter, then those with otherwise untreatable heroin addiction will be relegated to a world of unnecessary pain, forced to live at the bottom rung of society. There, they will be loathed for moral unevenness and imperfect personalities and doomed, by force of circumstance, to become a public menace and the target of law enforcement.

The addiction physician understands very well that illness is not only presented in the clinic; but instead originates and manifests itself most often in the everyday world of the addicted person and their family, a context where the physician may have little real influence. Nowhere is the social element of illness more unmistakable than in the life world of people living with addiction, and their families, where their social being is as much under threat by the tarnishing effects of a disparaging society as is their physical being by the hazards of unhealthy drugs and unhygienic needles. The person living with addictions is not alone in this position–there is a long history of illnesses that were stigmatized right out of medical practice but turned out to be quite treatable in the framework of modern medicine such as leprosy, schizophrenia, and today even AIDS. The only recourse is to thrust people living with addictions back into the medical realm and to support the practitioners who treat them humanistically.

This is not a new dilemma for medicine. In the Tate Gallery hangs a 19^th ^century canvas, simply titled "The Doctor". It depicts a small child stricken with illness–the limp body lays helplessly on a bed in a tiny cottage while a compassionate country doctor watches over the child the whole night until the early morning sun brings some resolution to the crisis and the child awakens restore to health by the passage of time – with little the doctor can do but be there for the child and the family.

The full breadth of the physician's work often ranges far beyond the "science" of treatment and disease – often inadequate to relieve suffering, and always at the wrong end of deaths final victory. But the compassionate presence of the doctor is a crucial part of the patient's healing journey, no matter how great the suffering and regardless of the ultimate outcome.

In the lived experience that inspired the work by Fildes's (1843–1927), the outcome was tragic – the child (the artists own son) did not live to see the morning sun and died Christmas morning 1877. But the kindness of the doctor so moved the father that he painted the canvas as an homage, not to medical science but to one doctors compassion and spirit of solidarity [51,52]. That is the true art in medicine. It is time for heroin prescription to become part of that art.
